# Genotypic and antimicrobial susceptibility of *Streptococcus agalactiae* causing bovine mastitis in the central region of Thailand

**DOI:** 10.3389/fvets.2023.1250436

**Published:** 2023-11-09

**Authors:** Sirirat Wataradee, Sukuma Samngamnim, Thanasak Boonserm, Kittisak Ajariyakhajorn

**Affiliations:** Department of Veterinary Medicine, Faculty of Veterinary Science, Chulalongkorn University, Bangkok, Thailand

**Keywords:** antimicrobial susceptibility, capsular polysaccharide, molecular epidemiology, multilocus sequence typing, random amplified polymorphic DNA

## Abstract

**Introduction:**

*Streptococcus agalactiae* is a highly contagious pathogen that causes bovine mastitis, leading to significant economic losses. This study aimed to (1) identify and characterize *S. agalactiae* strains responsible for bovine mastitis by examining their phenotypic and genotypic characteristics in Thai dairy-intensive farming areas and (2) determine their susceptibility profiles to antimicrobial agents.

**Material and methods:**

In total, 100 *S. agalactiae* isolates obtained from clinical and subclinical mastitis cases from 13 dairy herds located in the central region of Thailand were examined. To confirm the identity of the bacterial pathogens, conventional microbiological procedures recommended by the National Mastitis Council (NMC) and the VITEK^®^ 2 system were employed.

**Results:**

All 100 isolates were successfully identified as *S. agalactiae* using the NMC procedure, whereas 94 isolates were identified as *S. agalactiae* using the VITEK^®^ 2 system. Finally, the *S. agalactiae*-specific gene *dlt S* was identified in all the examined isolates using polymerase chain reaction. Capsular polysaccharide (CPS) typing revealed that all strains belonged to CPS type Ia. Multilocus sequence typing identified 33 selected isolates as sequence type 103. Random amplified polymorphic DNA (RAPD) typing yielded 43 RAPD types, with 6 RAPD clusters identified. These results demonstrated a high level of genetic diversity among *S. agalactiae* within the studied herds. RAPD analysis suggested that specific *S. agalactiae* strains could persist in dairy farms for 2–12 months. Furthermore, antimicrobial susceptibility testing was performed using the broth microdilution method. Most strains demonstrated susceptibility to ampicillin, penicillin, penicillin/novobiocin, cephalothin, oxacillin, ceftiofur, and erythromycin.

**Discussion:**

This study revealed the phenotypic and genotypic characteristics of *S. agalactiae* isolates responsible for bovine mastitis in the central region of Thailand. The rapid identification of *S. agalactiae* and application of molecular typing methods can provide valuable epidemiological information regarding *S. agalactiae* causing mastitis in dairy farms. The antimicrobial susceptibility of *S. agalactiae* indicates that antimicrobial treatment for control and eradication could be a successful protocol. Our findings revealed that a single clonal strain of *S. agalactiae* affected the 13 studied farms. Further research is needed to explore the feasibility of vaccine development and application.

## 1. Introduction

Mastitis is an inflammation of the udder caused by bacteria (1, 2). It poses a significant health concern and is a costly disease that affects dairy cows globally (3). In 2009, mastitis led to an annual economic loss of $2 billion in the USA (4). This problem also causes economic losses amounting to 500 million €, 3 billion €, and 125 billion € in Germany, the European Union, and worldwide, respectively (5). Mastitis-causing pathogens are generally classified into contagious and environmental pathogens (2). Highly contagious pathogens associated with mastitis include *Streptococcus agalactiae*, *Staphylococcus aureus*, and *Mycoplasma* spp. (6).

*Streptococcus agalactiae* is increasingly acknowledged as responsible for cow mastitis, newborn meningitis, and fish meningoencephalitis. It remains a common cause of bovine mastitis in emerging dairy industries (7). Furthermore, *S. agalactiae* is among the top two pathogens contributing to bovine mastitis in Southeast Asia, leading to both clinical and subclinical cases (8). This bacterium impairs mammary secretory cells, resulting in reduced milk production, which, in turn, impacts the economic viability of farms (9). An *S. agalactiae*-infected cow negatively affects bulk tank milk somatic cell count (SCC), thereby increasing SCC and subsequently reducing the raw milk quality for processing and shortening the shelf life of dairy products (3, 10, 11). Affected cows also exhibit a high level of lipolytic and proteolytic enzymes in milk, which further affects the milk’s shelf life and taste (12, 13).

*S. agalactiae* intramammary (IMM) infection is highly contagious and rarely resolves spontaneously. The use of beta-lactam antimicrobials is the recommended treatment strategy for *S. agalactiae* mastitis (9, 14). Consequently, a significant quantity of antimicrobials has been utilized in the treatment of *S. agalactiae*-infected cattle. Thai dairy farming areas are situated in tropical regions that are endemic to diseases such as foot and mouth disease and various blood parasites. Health concerns related to these diseases and their complications often necessitate the use of antimicrobials for therapeutic purposes. The widespread use of antimicrobial drugs is prevalent in Thailand for the treatment of common infectious diseases such as bacterial complications of foot and mouth disease (15), respiratory infections like bovine respiratory disease complex (16), gastrointestinal infections, blood parasite infections, reproductive tract infections, and mastitis. Common IMM infusion antimicrobials used for mastitis treatment in Thailand include penicillin, ampicillin, cloxacillin, cephalosporins, gentamicin, neomycin, and tetracycline. In cases where intramuscular administration is required for mastitis treatment, several antimicrobials are available, including amoxicillin, cephalosporins, gentamicin, and tetracycline. The improper use of antimicrobials has contributed to the development of antimicrobial resistance among microorganisms found in animals and the environment (17). Therefore, surveillance studies that focus on antimicrobial susceptibility are essential to promote responsible antimicrobial use and mitigate the risk of future resistance emergence.

An epidemiological study is crucial to explore the genetic diversity, transmission routes, and distribution of *S. agalactiae* strains in circulating areas and ultimately develop effective strategies to prevent and control the dissemination of this mastitis-causing pathogen (18). Capsular polysaccharide (CPS) genotyping is a commonly employed molecular epidemiological tool for the investigation of mastitis-causing *S. agalactiae*. Variations of the CPS structure are closely linked to the genetic diversity of the polysaccharide capsule of the pathogen (19–21). Within-herd investigations often utilize comparative methods such as pulsed-field gel electrophoresis (PFGE), amplified fragment length polymorphism, and random amplified polymorphic DNA (RAPD) analysis of genomic DNA. Among these methods, RAPD analysis has emerged as a rapid and straightforward DNA-based typing approach with high discriminatory power, enabling the differentiation of strains within each cluster (20, 22, 23). RAPD typing has demonstrated sufficient discriminatory power (*D >* 0.90) and acceptable reproducibility when analyzing isolates simultaneously (7). Furthermore, multilocus sequencing (MLST) data are currently employed to examine the population dynamics and molecular genetic evolution of *S. agalactiae* (14, 24, 25). MLST is advantageous for large-scale comparisons and allows geographical differentiation. A comprehensive understanding of the epidemiology of this mastitis-causing pathogen can provide valuable insights into the control of bovine mastitis caused by *S. agalactiae* in dairy herds. Based on epidemiological studies conducted in China and Thailand, CPS type Ia has emerged as the most prevalent type in Asia (26, 27). In the northern region of Thailand, 17 isolates of *S. agalactiae* causing bovine mastitis were identified as a single CPS type Ia and sequence type (ST) 103 strain (28).

Since 1962, Thailand’s dairy sector has undergone significant growth. The majority of dairy herds in the country are managed by small-scale producers or small dairy holders (29). As of 2022, these farms have approximately 760,000 lactating cows (30). The central region of Thailand is a prominent center for intensive dairy farming, with Saraburi, Nakhon Ratchasima, and Ratchaburi ranking as the top three provinces, accounting for 44.26% of the total dairy cattle population in the country in 2021 (31). However, only few studies have focused on bovine mastitis caused by *S. agalactiae* in Thailand. Existing studies have revealed a high prevalence of the pathogen in bulk tank milk at the herd level, with 46.30% in Chiang Mai province (8). The prevalence of *S. agalactiae* in cases of bovine mastitis was 17% at the cow level in the northern part (32) and 21.8% at the herd level in Khon Kaen province (33). Consequently, no studies have examined the epidemiology of *S. agalactiae* strains found in farms in Thailand, particularly in the central regions.

Therefore, this study primarily aimed to identify and characterize the *S. agalactiae* strains responsible for bovine mastitis in Thai dairy-intensive farming areas, focusing on their phenotypic, and genotypic characteristics, and to investigate the antimicrobial susceptibility profiles of these strains.

## 2. Materials and methods

### 2.1. Ethics approval statement

All procedures have been reviewed and approved by the Institutional Biosafety Use protocol (Chulalongkorn University, Faculty of Veterinary Science) No. IBC 1931051.

### 2.2. Bacterial isolates and sample collection

In total, 100 *S. agalactiae* isolates were obtained from 58 lactating cows diagnosed with clinical (*n* = 21) and subclinical (*n* = 37) mastitis between 2016 and 2019. Clinical mastitis cases were assessed by a veterinarian through visual examination, which involved evaluating milk abnormalities and udder abnormalities including warmth, redness, and swelling. Subclinical mastitis was determined using the California mastitis test, resulting in positive findings. Quarter culture analysis led to the identification of *S. agalactiae* in one quarter (*n* = 33), two quarters (*n* = 11), three quarters (*n* = 11), and all quarters (*n* = 3) of infected cows (Table 1). These mastitis cases were reported from 13 dairy farms located in the provinces of Saraburi (14° 31′ 25.79″ N, 100° 54′ 24.59″ E), Nakhon Ratsima (14° 58′ 14.38″ N, 102° 06′ 7.06″ E), and Ratchaburi (13° 32′ 12.16″ N, 99° 49′ 1.63″ E) (Table 1).

**Table 1:** Characteristics of the 100 *S. agalactiae* isolates associated with mastitis.

Herd	Province	Year	Herd size^a^	Number of isolates	Number of cases	Number of clinical cases	Number of subclinical cases	One quarter infected (cow)	Two quarters infected (cow)	Three quarters infected (cow)	All quarters infected (cow)
Total isolates/cow				100	58	21	37	33	11	11	3
A	Saraburi	2018–2019	L	31	22	12	10	16	4	1	1
B	Nakorn-Ratsima	2016	M	1	1	0	1	1			
C	Saraburi	2017	S	1	1	0	1	1			
D	Nakorn-Ratsima	2016–2017	M	9	4	0	4	1	1	2	
E	Saraburi	2018	S	1	1	1	0	1			
F	Nakorn-Ratsima	2016–2017	M	18	6	2	4	1		3	2
G	Nakorn-Ratsima	2017	S	3	3	0	3	3			
H	Saraburi	2017	M	3	2	0	2	1	1		
I	Saraburi	2017	S	8	3	0	3		1	2	
J	Saraburi	2016	M	15	8	2	6	3	3	2	
K	Saraburi	2017	M	4	2	0	2	1		1	
L	Ratburi	2019	L	5	4	4	0	3	1		
M	Nakorn-Ratsima	2019	M	1	1	0	1	1			

a Herd size, average number of lactating cows per herd during the study period; L, large, >100 dairy cows; M, medium, 21–100 dairy cows; S, small, <20 dairy cows.

All isolates included in this study were previously identified as *S. agalactiae* following the guidelines outlined in the National Mastitis Council (NMC) Laboratory Handbook on Bovine Mastitis (24). The strains were identified by the Microbiology Diagnostic Unit of the Ruminant Medicine Division, Department of Veterinary Medicine, Faculty of Veterinary Science, Chulalongkorn University. The isolates have been securely stored at −80°C for further analysis. The study was conducted in Room 2001005547, a biosafety level (BSL) 2 laboratory located at the Livestock Hospital within the Faculty of Veterinary Science at Chulalongkorn University in Nakorn Pathom province, Thailand.

The isolates were retrieved from a −80°C freezer stock using the culture-based method, involving subculturing on sheep blood agar media twice before testing. In brief, samples stored at −80°C were allowed to reach room temperature. Subsequently, the samples were streaked on blood agar plates using a sterile inoculating loop (streak plate method). The sample plates were incubated with carbon dioxide at 37°C overnight. After incubation, a pure colony was observed on the plate. Subsequently, it was subcultured for the second round, and a single pure colony was selected for further analysis.

### 2.3. Reference strains

The following bacterial reference strains were obtained from the American Type Culture Collection (ATCC; LGC Standards), which were used for laboratory quality control: *S. aureus* ATCC 25923 (CAMP test, positive control for the catalase test) and *S. agalactiae* ATCC 12400 (CPS type Ia), ATCC 13813 (CPS type II), and ATCC 31475 (CPS type III).

### 2.4. DNA template preparation for molecular procedures

DNA extraction was performed in a BSL-2 cabinet. DNA was extracted from the pure culture using DNeasy^®^ Blood & Tissue Kit (Qiagen, Hilden, Germany) according to the instruction protocol. The concentration and purity of the extracted DNA were measured using a spectrophotometer (Nanodrop^™^ Lite, Thermo Fisher Scientific^™^, MA, United States). The extracted DNA was used as the template for all polymerase chain reaction (PCR) assays. All DNA templates were collected and stored at −20°C for further analysis.

### 2.5. Identification tools

The 100 isolates were identified using three methods: (1) conventional microbiological techniques following the NMC Laboratory Handbook on Bovine Mastitis (2). Briefly, all isolates were identified based on their colony morphology, hemolysis type, esculin hydrolysis, catalase production, and CAMP reaction; (2) automated microbial identification using the VITEK^®^ 2 system (bioMérieux, Marcy-l’Etoile, France) following the manufacturer’s protocol; and (3) conventional genotypic molecular PCR using specific primers for the *dlt s* gene (34, 35). The primers *dlts*-F (5′-AGGAATACCAGGCGATGAACCGAT-3′) and *dlts*-R (5′-TGCTCTAATTCTCCCCTTATGGC-3′) were used. PCR was performed following the protocol used by Poyart et al. (34). Each PCR mixture (25 μL) contained 12.5 μL of 10× Green GoTaq^®^ Flexi Buffer (PROMEGA, Madison, WI, United States), 1 μL of the forward primer, 1 μL of the reverse primer, 2 μL of the DNA template, and 8.5 μL of distilled water (DW). The PCR mixtures underwent amplification in a DNA thermal cycler (T100 Thermal cycler, Bio-Rad Laboratories, CA, United States^®^), involving an initial denaturation step for 5 min at 95°C, followed by 35 cycles at 95°C for 1 min, 55°C for 1 min, 72°C for 2 min, and a final extension cycle at 72°C for 5 min. The PCR products and a 1-kb DNA size marker were analyzed in a 1.5% agarose gel, stained with nucleic acid gel stains (GelRed^®^, Geneon) at 100 V for 1 h. Finally, the agarose gel was visualized and photographed using a UV transilluminator. To confirm the identification of *S. agalactiae*, a PCR amplicon size of 952 bp was expected.

### 2.6. Molecular characterization: CPS typing, MLST, and RAPD typing

Molecular characterization assays, including CPS, MLST, and RAPD fingerprint typing, were employed to assess the genotypic diversity and relationships among the isolates.

#### 2.6.1. CPS typing

The CPS type of the studied isolates was determined through PCR assays targeting nine *cps* genes. This technique was previously introduced by Poyart et al. (34). The sequence of CPS type-specific primers and the predicted amplicon sizes are shown in Table 2. The PCR reaction mixture was prepared by adding 12.5 μL of 2× Green GoTaq^®^ Flexi Buffer (PROMEGA), 1 μL of the forward primer, 1 μL of the reverse primer, 2 μL of the DNA template, and 8.5 μL of DW. In the case of a negative control, 10.5 μL of DW was used. Positive controls included the reference *S. agalactiae* strains ATCC 12400 (type Ia), ATCC 13813 (type II), and ATCC 31475 (type III). The PCR mixtures were amplified in a DNA thermal cycler (T100^™^ Thermal Cycler, Bio-Rad Laboratories) with an initial denaturation step for 5 min at 95°C, followed by 35 cycles at 95°C for 1 min, 55°C for 1 min, 72°C for 2 min, and a final extension cycle at 72°C for 5 min. The PCR products were analyzed on a 1.5% agarose gel, stained with nucleic acid gel stain (GelRed^®^, Geneon), and subjected to gel electrophoresis at 100 V for 1 h. Finally, the agarose gel was visualized and photographed using a UV transilluminator.

**Table 2:** CPS type-specific primers and prediction of PCR products based on computer simulation data from Poyart et al. (34).

Primer name	Sequences (5′–3′)	Gene targets	Amplicon sizes (bp)
Ia-F	GGTCAGACTGGATTAATGGTATGC	*cps1aH*	521 and 1,826
Ia-R	GTAGAAATAGCCTATATACGTTGAATGC	*cps1aH*	
Ib-F	TAAACGAGAATGGAATATCACAAACC	*cps1bJ*	770
Ib-R	GAATTAACTTCAATCCCTAAACAATATCG	*cps1bK*	
II-F	GCTTCAGTAAGTATTGTAAGACGATAG	*cps2K*	397
II-R	TTCTCTAGGAAATCAAATAATTCTATAGGG	*cps2K*	
III-F	TCCGTACTACAACAGACTCATCC	*cps1a/2/3I*	1,826
III-R	AGTAACCGTCCATACATTCTATAAGC	*cps1a/2/3 J*	
IV-F	GGTGGTAATCCTAAGAGTGAACTGT	*cps4N*	578
IV-R	CCTCCCCAATTTCGTCCATAATGGT	*cps4N*	
V-F	GAGGCCAATCAGTTGCACGTAA	*cps5O*	701
V-R	AACCTTCTCCTTCACACTAATCCT	*cps5O*	
VI-F	GGACTTGAGATGGCAGAAGGTGAA	*cps6I*	487
VI-R	CTGTCGGACTATCCTGATGAATCTC	*cps6I*	
VII-F	CCTGGAGAGAACAATGTCCAGAT	*cps7M*	371
VII-R	GCTGGTCGTGATTTCTACACA	*cps7M*	
VIII-F	AGGTCAACCACTATATAGCGA	*cps8J*	282
VIII-R	TCTTCAAATTCCGCTGACTT	*cps8J*	

#### 2.6.2. MLST

Thirty-three isolates were selected for MLST to represent both clinical and subclinical mastitis cases from all the studied herds. Therefore, at least one isolate per clinical and subclinical mastitis case within a herd was selected. Additionally, isolates from repeated mastitis cases were included (Supplementary Table S1).

MLST for *S. agalactiae* was performed as described by Jones et al. (36). Nested PCR, using a combination of two primers (amplification primer and sequencing primer) in one reaction, was conducted with the primers *adhP*, *pheS*, *atr*, *glnA*, *sdhA*, *glcK*, and *tkt*. All the generated sequences were assigned an allele number and combined into an allelic profile. The PubMLST database (www.pubmlst.org) was used to determine the ST. A comparative analysis of geographic regions based on ST, using the eBURST program, led to the designation of clusters or clonal complexes.

#### 2.6.3. RAPD typing

RAPD fingerprints were determined according to the protocol described by Martinez et al. (22). The reaction was performed in triplicate to ensure laboratory quality control. RAPD typing was replicated using the same DNA extraction, and RAPD typing was conducted independently to ensure reproducible results. The entire PCR mixture (25 μL) included 12.5 μL of 10× Green GoTaq^®^ Flexi Buffer (PROMEGA), 0.4 μM of primer, and 50 ng of DNA template. Primers for OPS-11 (5′-AGTCGGGTGG-3′) were used in this study. The PCR mixtures were subjected to a DNA thermal cycler (T100 Thermal Cycler, Bio-Rad^®^), beginning with an initial denaturation step for 5 min at 94°C, followed by 35 cycles at 94°C for 30 s, 55°C for 1 min, 72°C for 5 min, and a final extension cycle at 72°C for 5 min. The reference *S. agalactiae* ATCC 13813 strain was used as a control, and DW was used for the negative control. All amplified products and a 1-kb DNA size marker were analyzed in a single batch on a 2% agarose gel, stained with nucleic acid gel stain (GelRed^®^, Geneon), at 70 V for 180 min. Finally, the agarose gel was visualized and photographed using a UV transilluminator.

The similarities and differences among the isolates were analyzed based on a binary matrix. Densitometric analysis, normalization of densitometric traces, and interpolation of the profiles were conducted using the DICE coefficient and the unweighted pair group method with arithmetic mean to generate the dendrogram based on average linkage. This dendrogram was used to determine the relatedness of *S. agalactiae* isolates. Isolates with ≥80% similarity were considered to belong to the same genotypic pattern or cluster. The discriminatory power (D) was calculated using the Hunter–Gaston formula (37).


$$D=1-\frac{1}{N\left(N-1\right)}\sum_{j=1}^{s}X_{j}\left(X_{j}-1\right)$$

In this formula, *N* is the total number of isolates in the study population, *s* is the total number of different pattern types, and *X_j_* is the number of isolates belonging to the *j*^th^ type.

### 2.7. Antimicrobial susceptibility of *Streptococcus agalactiae* isolates

Antimicrobial susceptibility was assessed using the broth microdilution method. Minimum inhibitory concentrations (MICs) were determined through broth microdilution, employing the semi-automatic Sensititre™ system from Thermo Fisher Scientific^™^. The procedure adhered to the guidelines provided in the Thermo Scientific Sensititre^™^ plate guidelines for antimicrobial susceptibility testing. A Sensititre^™^ standard susceptibility mastitis plate (CMV1AMAF; Sensititre^™^, TREK Diagnostic Systems, LLC, Cleveland, OH, United States) was used. The CMV1AMAF panel comprised the following 10 antimicrobials in a serial two-fold dilution: ampicillin, ceftiofur, cephalothin, erythromycin, oxacillin, penicillin/novobiocin combination, pirlimycin, sulfadimethoxine, and tetracycline. The range of concentrations of antimicrobials used in the CMV1AMAF plate is shown in Table 3. *S. aureus* ATCC 29213 was employed as the laboratory control strain. MIC was defined as the lowest concentration of an antimicrobial that inhibited the visible growth of an isolate. The MIC breakpoints (μg/mL) were derived from the Clinical and Laboratory Standards Institute (CLSI) guidelines for *S. agalactiae* (CLSI 2019 and 2020) supplement VET08 ED4 or M100 ED30. The MIC breakpoints were interpreted as susceptible, resistant, and intermediate according to CLSI VET08. MIC_50_ and MIC_90_ represent the concentrations at which 50 and 90% of the studied isolates were inhibited, respectively.

**Table 3:** Range of concentrations of antimicrobials used in the CMV1AMAF plate.

Antimicrobial	Concentration range (ug/mL)
Ampicillin	0.12–8
Ceftiofur	0.5–4
Cephalothin	2–16
Erythromycin	0.25–4
Oxacillin	2–4
Penicillin	0.12–8
Pirlimycin	0.5–4
Penicillin/novobiocin	Penicillin (1–8)/novobiocin (2–16)
Sulfadimethoxine	32–256
Tetracycline	1–8

## 3. Results

### 3.1. Isolate identification

All 100 isolates were assessed for their phenotypic characteristics, including the presence of beta hemolysis on blood agar for bacterial colony growth (*n* = 88). Furthermore, all isolates produced positive results in the CAMP test but negative results in the esculin hydrolysis and catalase tests.

The VITEK^®^ 2 identification system successfully identified 94 isolates as *S. agalactiae*; 2 isolates were misidentified as *S. canis*, while 4 were not identified. The probability of identification classification, based on the percentage of probability, indicated a good-to-excellent probability of identification at 93.61% (88 out of 94). Using the VITEK^®^ 2 system, the identification time for *S. agalactiae* ranged from 4.60 to 7.80 h.

All 94 identified *S. agalactiae* isolates exhibited positive reactions in the fermentation and acid production tests, including d-galactose, lactose, d-maltose, d-mannose, N-acetyl-d-glucosamine, lactose, phosphatase, and alanine arylamidase. Miscellaneous tests such as polymyxin, bacitracin, and novobiocin also yielded positive results. However, the *S. agalactiae* isolates tested negative in the fermentation and acid production tests for d-amygdalin, d-xylose, d-sorbitol, d-mannitol, d-raffinose, phosphatidylinositol phospholipase C, l-aspartate arylamidase, beta-galactopyranosidase, alpha-mannosidase, l-pyroglutamyl arylamidase, and urease.

Furthermore, the 94 *S. agalactiae* isolates were classified into 37 bionumber types, with a discriminatory power of 0.915. Among the isolates identified using this system, the majority belonged to bionumber type 1 (*n* = 25) (data not shown).

Genotypic identification using the specific *dlt S* gene confirmed that all the studied isolates belong to *S. agalactiae*. This confirmation was achieved through three bacterial identification procedures, all of which confirmed that our studied isolates were indeed *S. agalactiae*.

### 3.2. Genotype characterization via CPS typing, MLST, and RAPD typing

#### 3.2.1. CPS typing and MLST of *Streptococcus agalactiae*

All isolates were classified as CPS type Ia (*n* = 100). In the MLST study involving the 33 selected isolates, only one multilocus ST (ST 103) was identified in all 13 herds, indicating a consistent correlation between the CPS and MLST genotypes and confirming the existence of a single *S. agalactiae* clone in our study.

#### 3.2.2. RAPD typing

The OPS-11 primer employed in the typing method for RAPD fingerprinting showed that consistently yielded identical band patterns in triplicate reactions (data not shown). The resulting RAPD fingerprints exhibited the varied number of bands, ranging from 3 to 7, with sizes spanning 300–2,200 bp. A dendrogram was constructed based on these RAPD profiles, and a similarity analysis revealed 43 distinct RAPD types among the 100 *S. agalactiae* isolates (Supplementary Figure S1).

The discriminatory power of RAPD typing was 0.979, indicating its effectiveness in distinguishing between isolates. By applying a similarity threshold of ≥80%, the 43 RAPD types were further classified into six clusters. The major cluster groups were C and E, which encompassed 12 and 11 RAPD types, respectively, accounting for 36% (*n* = 34) and 32% (*n* = 30) of the studied isolates (Supplementary Table S2).

The distribution of RAPD cluster types from 2016 to 2019 in our collection of *S. agalactiae* isolates revealed that clusters C (*n* = 34), D (*n* = 12), E (*n* = 30), and F (*n* = 9) were consistently identified throughout this period. Clusters A and B (*n* = 5) were observed in 2016 and 2019 only (Supplementary Figure S2).

##### 3.2.2.1. Within-farm genotype relatedness of *Streptococcus agalactiae*

According to RAPD typing, *S. agalactiae* isolates were classified into six clusters: A, B, C, D, E, and F, with each cluster containing varying numbers of subclusters (3, 4, 12, 5, 11, and 3, respectively). Subclusters grouped pathogens with 100% identical RAPD types, indicating a close genetic relationship.

In two subcluster groups, isolates were obtained from the same cow but different quarters during specific periods. In herd I, subcluster E35.1 was isolated from the left front quarter of a cow named “dangnoi,” while subcluster E35.2 was isolated from the left hind quarter of the same cow. Similarly, in herd F, subcluster F37.1 was isolated from the right front quarter of a cow named “aumpun,” and subcluster F37.2 was isolated from the left hind quarter of the same cow.

Furthermore, one subcluster was able to infect at least two cows simultaneously in several herds (A [C9.3 and C9.4], J [A1.1 and A1.2], and F [F37.2 and F37.3]).

Several subclusters, including C13.1–C13.3, C16.1–C16.3, and C18.1–C18.3, were able to cause mastitis occurrences for up to 2–7 months. In herd F, subcluster E (specifically E29.1 and E29.3) was observed, with two cows exhibiting an association with these subclusters over 6 months. Similarly, in herd D, subcluster C (comprising C11.1 and C11.3) was identified, with two cows showing an association with these subclusters over 12 months.

Among the 14 mastitis cases, the cows had ≥2 quarters infected by *S. agalactiae*. RAPD genotype clusters may be related to the VITEK^®^ 2 bionumber phenotype. Only the following six cases demonstrated a relationship between the RAPD cluster and VITEK^®^ 2 bionumber type: cow “5543” (RAPD clusters C13.1 and C18.2 related to VITEK^®^ 2 bionumber type 1), cow “58007” (clusters E26.1 and E27.2 related to bionumber type 1), cow “58041” (clusters C16.2, C18.3, and C9 related to bionumber type 1), cow “gene” (clusters E31.1 and E31.2 related to bionumber type 1), cow “jumba” (clusters E31.1 and E31.2 related to bionumber type 1), and cow “som” (clusters C10.3 and C14 related to bionumber type 20) (Supplementary Table S4).

### 3.3. Antimicrobial susceptibility of *Streptococcus agalactiae*

The 100 *S. agalactiae* isolates obtained from 2016 to 2019 were tested against 10 antimicrobial drugs. Table 4 displays the MIC ranges for each antimicrobial agent, along with their corresponding MIC_50_ and MIC_90_ values. Both MIC_50_ and MIC_90_ for ampicillin, penicillin, penicillin/novobiocin, cephalothin, ceftiofur, erythromycin, oxacillin, and pirlimycin were 0.125, 0.125, 1/2, 2, 0.5, 0.25, 2, and 0.5 μg/mL, respectively. The MIC_50_ and MIC_90_ for tetracycline were 1 and 8 μg/mL, respectively, whereas those for sulfadimethoxine were 128 and 256 μg/mL, respectively.

**Table 4:** Minimum inhibitory concentrations (MICs) (μg/mL) with representative susceptible phenotypes^a^ of *Streptococcus agalactiae* strains (isolates, *n* = 100).

Antimicrobial agents	MIC_50_^b^	MIC_90_^b^	Number of isolates at each indicated MIC (μg/mL)^a^											
			0.125	0.25	0.5	1	2	4	8	16	32	64	128	256
Ampicillin	0.125	0.125	100											
Penicillin	0.125	0.125	99					1						
Penicillin/novobiocin^c^	1/2	1/2				100								
Cephalothin	2	2					100							
Ceftiofur	0.5	0.5			98	1		1						
Erythromycin	0.25	0.25		99				1						
Oxacillin +2% NaCl	2	2					100							
Pirlimycin	0.5	0.5			100									
Tetracycline	1	8				65	1	1	33					
Sulfadimethoxine	128	256									41	7	2	50

a The MIC breakpoints (μg/mL) were derived from the Clinical and Laboratory Standards Institute (CLSI) guidelines for *S. agalactiae* (CLSI 2019 and 2020) supplement VET08 ED4 or M100 ED30. The light and darker gray shades represent the susceptible and resistant zones, respectively.

b MIC_50_ and MIC_90_: MIC (μg/mL) that inhibited 50 and 90%, respectively, of the isolates.

c Concentration of combination drug: penicillin (1–8 μg/mL)/novobiocin (2–16 μg/mL).

All isolates exhibited susceptibility to ampicillin, penicillin/novobiocin, cephalothin, oxacillin, and pirlimycin. Moreover, 99% of the isolates were susceptible to penicillin, followed by erythromycin (99%) and ceftiofur (98%). Notably, *S. agalactiae* isolates were resistant to tetracycline (33%) and sulfadimethoxine (50%).

## 4. Discussion

This study identified and characterized *S. agalactiae*, which causes bovine mastitis among animals in dairy-intensive farming areas in the central region of Thailand. Knowledge on mastitis caused by this pathogen is useful for the application of effective control and prevention strategies.

*S. agalactiae*, commonly known as Group B streptococcus, is a causative pathogen of bovine mastitis and plays a crucial role in the control and eradication of IMM infections caused by *S. agalactiae*. The standard method for diagnosing *S. agalactiae* involves bacterial culture of milk samples on blood agar, following the procedure established by the NMC (24, 38). This method is cost-effective, easy to perform, and highly accurate in general microbiology laboratories. However, its detection sensitivity ranges from 20.5 to 78% (38), while the specificity of individual bacterial culture is nearly 100% (39). VITEK^®^ 2, a semi-automated bacterial identification system, facilitates rapid identification, with results available within 3–8 h. The system generates a report based on biochemical reaction patterns. However, there are some limitations to this method, including the need for a high concentration of pure colonies and a limited dataset in the cloud to differentiate pathogens (40). Conventional molecular PCR targets the *S. agalactiae*-specific *dlt S* gene for exclusive identification (35). It has a high specificity and sensitivity and can identify organisms at the genetic level (41). The choice of identification systems depends on resource availability and time constraints. Each identification method has its strengths and limitations, and their selection is based on various factors, such as resource availability and the specific application purpose. A combination of these methods can potentially be used to achieve accurate and rapid testing, aiding decision-making and the selection of the most suitable approach for specific objectives.

Our findings demonstrated that both the conventional NMC method and molecular PCR, using the *S. agalactiae*-specific gene *dlt S*, accurately identified 100% of the *S. agalactiae* strains. We explored the potential applications of these methods in clinical and veterinary settings. The VITEK^®^ 2 system exhibited 94% agreement with the conventional NMC method. However, it is important to note that the conventional microbiological approach currently serves as the standard procedure for identifying mastitis-causing pathogens, especially when dealing with milk samples. This method involves laborious preparation, using agar and various biochemical reagents, and has inherent limitations in terms of sample testing capacity and time efficiency, with a minimum turnaround time of 48 h. The VITEK^®^ 2, a semi-automated bacterial identification system, is designed to address the limitations of conventional microbiological methods. It successfully identified 94% of *S. agalactiae* strains causing mastitis in our study, consistent with another study on gram-positive cocci identification where 90% of the isolates were identified (40). Additionally, 96.5% of *S. agalactiae* strains from human clinical isolates were correctly identified by VITEK^®^ 2 (42). It is noteworthy that the identification of *S. agalactiae* strains can also be achieved by leveraging various biochemical enzymatic activities, opening up potential avenues for the development of rapid identification test kits.

The *dlt S* gene is specific to *S. agalactiae*. PCR detection of this gene is beneficial for detecting both live and dead microorganisms. This molecular marker has found widespread use in identifying *S. agalactiae* isolates in several studies (34, 35, 43). The use of the 16S rRNA gene has been used to identify *S. agalactiae* in humans with an efficiency rate of 85.71% (44). Additionally, real-time multiplex PCR, incorporating the *dlt S* gene and capsular typing genes, can rapidly identify this pathogen in human infections (45). The molecular PCR method, using the *S. agalactiae-*specific *dlt S* gene for identification, has gained attention owing to its advantages, including reduced time consumption and high accuracy in detecting *S. agalactiae* in blood samples. In the future, this method could be applied to detect *S. agalactiae* in mastitis milk samples.

As a result, the conventional microbiological method remains the standard for *S. agalactiae* identification in many laboratories. The VITEK^®^ 2 system is capable of facilitating rapid identification with multiple samples. The conventional genotypic molecular PCR, utilizing the specific *dlt S* gene, has exceptional accuracy and rapidity. Currently, matrix-assisted laser desorption/ionization time-of-flight mass spectrometry, commonly abbreviated as MALDI-TOF MS, is an intriguing tool for identification and characterization (46, 47). It has been employed in milk quality laboratories, serving a dual role as a diagnostic tool and a research facilitator, primarily focusing on the identification of bacterial species (48). Although there have been no reports on MALDI-TOF MS usage in identifying *S. agalactiae*, a previous study on *Streptococcus uberis* aimed to identify potentially contagious strains and predict the clinical risk of mastitis in cows; that study revealed the ability to predict the risk of new clinical mastitis transmission in herds infected with *S. uberis* (49).

Previous research on the genotypic diversity and distribution of *S. agalactiae* in dairy herds and regional dairy populations has provided valuable epidemiological data. CPS genotyping and MLST have become universal approaches for comparative typing, especially suited for large-scale comparisons (14). Several studies have employed CPS typing and MLST to investigate the epidemiology of *S. agalactiae* in dairy animals (14, 20, 22, 50, 51). In the present study, CPS genotyping was used as a molecular epidemiological tool to explore *S. agalactiae* causing bovine mastitis in 13 Thai dairy farms. All *S. agalactiae* strains were identified as CPS type Ia and ST 103. Our findings revealed that this clone of *S. agalactiae* predominated in the evaluated dairy farms, consistent with the results of a previous study conducted in northern Thailand (28). The distribution of *S. agalactiae* capsular types associated with bovine mastitis varies globally, with specific types prevailing in particular geographic regions. For instance, in China, CPS genotypes Ia and II are the most prevalent (51). In Asia, CPS type Ia is the most frequently reported type, as observed in studies conducted in Thailand and China (26–28). However, the CPS types of *S. agalactiae* isolates exhibit diversity in several countries, including Denmark (CPS types Ia, Ib, and V), Finland (types Ia, Ib, II, and IV), Brazil (types Ia, Ib, II, III, and IV), Germany (types Ia, Ib, and III), and Iran (types II and III) (25, 35, 52–54).

In the present study, MLST analysis of 33 selected strains revealed the presence of ST 103 exclusively. Our findings did not provide evidence of population dynamics or molecular genetic evolution within *S. agalactiae* in this area. However, our study was conducted in the central region of Thailand, which is known for its high-density farming areas, and revealed a significant genetic similarity among *S. agalactiae* isolates from herds. This finding suggests the potential transmission of a dominant clone, likely due to inadequate adoption of within-herd biosecurity measures and cow-to-cow transmission during milking (38). Understanding the prevalent genotypes within populations is crucial for effectively preventing and controlling *S. agalactiae* infections. In terms of clinical implications, CPS type Ia and ST 103 can be considered promising candidates for vaccine development tailored to the study area. These findings provide valuable insights for the design of targeted vaccines, particularly for further autologous vaccine development.

Several studies have highlighted the genetic diversity revealed via RAPD typing in cases of *S. agalactiae* causing bovine mastitis. RAPD typing is a cost-effective and rapid DNA-based typing method with high discriminatory power, enabling strain differentiation and determination of genetic relationships within study samples (20, 22, 23, 55). In this study, the RAPD technique demonstrated a high level of reproducibility, as evidenced by consistent amplification of the same DNA band pattern in three separate examinations (data not shown). RAPD fingerprinting identified a total of 43 RAPD types, which were subsequently classified into six cluster groups (A–F) using an 80% similarity threshold. When RAPD typing was combined with OPS-11 primer and CPS serotyping, a previous study found that the combined method exhibited high discriminatory power (*D =* 0.95) (55). Another study showed that RAPD typing had high discriminatory power, similar to that of PFGE (50). However, PFGE has limitations in typing *S. agalactiae*, as it is associated with typing failures in isolates from the same herd (54).

In this study, the subclusters of *S. agalactiae* demonstrated genetic relatedness among infected quarters within the same cow, suggesting a pattern of pathogen transmission. This finding also implies contagious behavior, indicating the ability of the pathogen to transmit from cow to cow within the same herd and to circulate within the herd for extended periods when control and prevention strategies may be less effective. The occurrence of direct transmission between mammary glands during the milking procedure serves as a strong indicator of the effectiveness of the biosecurity program implemented in infected herds, as supported by a previous study (39).

The MIC value is essential information in clinical microbiology for determining the appropriate antimicrobial concentration levels for treatment and for monitoring antimicrobial resistance patterns in the studied area. In our study, eight antimicrobials, including ampicillin, penicillin, penicillin/novobiocin, cephalothin, ceftiofur, erythromycin, oxacillin, and pirlimycin, inhibited *S. agalactiae* at the lowest tested concentrations. In Thailand, the available IMM antimicrobials are penicillin, ampicillin, cloxacillin, gentamicin, cephalothin, cefuroxime, and ceftiofur. Based on our results, penicillin, ampicillin, cephalothin, and ceftiofur are recommended for the treatment of *S. agalactiae* in the evaluated area. However, it is important to note that ceftiofur is a third-generation cephalosporin used to treat refractory conditions in both human and veterinary medicine. Its use is restricted to veterinarian prescription, following the practical guidelines for veterinary practice in New Zealand (56). Therefore, antimicrobials should only be selected and prescribed by a veterinarian to promote the responsible use of antimicrobial agents on dairy farms.

In a study conducted in Brazil from 2014 to 2015, bovine *S. agalactiae* isolates showed high susceptibility to ampicillin, penicillin, cephalothin, and ceftiofur, while the rate of resistance to tetracycline was notably high (31.4%) (23). The reduced effectiveness of tetracycline against *S. agalactiae* has been attributed to its prior excessive use as an antimicrobial agent (57). Inappropriate antimicrobial use in dairy farming can lead to excessive utilization, incorrect selection of antimicrobials, improper dosing, and failure to follow recommended withdrawal periods. Several key factors contribute to the widespread improper use of antimicrobials, including inadequate regulation, a lack of veterinary oversight, and limited education and awareness. It is essential to emphasize the seriousness of this situation because inappropriate antimicrobial usage in dairy farming significantly contributes to the development and dissemination of antimicrobial resistance.

The treatment approach should involve the identification and treatment of all infected quarters with appropriate antimicrobials. IMM antimicrobial treatment has shown a high success rate in managing mastitis caused by *S. agalactiae*. In a previous study, IMM treatment of *S. agalactiae* with cefquinome achieved a high cure rate in 14–21 days. However, in cases without antimicrobial treatment, new infections were reported in the initially negative culture or previously uncured quarters group. Furthermore, the untreated quarters exhibited high levels of *S. agalactiae* shedding (58).

To minimize antimicrobial use in dairy farms, potential alternatives must be explored. Antimicrobial peptides obtained from natural sources or synthetic peptides (such as Pm11) have shown significant antibacterial activity against both gram-positive and gram-negative bacteria associated with bovine mastitis in Thailand, including *S. agalactiae*, *S. uberis*, *S. aureus*, and *Escherichia coli* (59). Further studies are needed to explore IMM treatment strategies and IMM prototype products.

To the best of our knowledge, this study investigated *S. agalactiae* as the causative agent of mastitis in dairy-intensive farming areas in Thailand. We elucidated both the phenotypic and genotypic characteristics of *S. agalactiae* isolates responsible for bovine mastitis. Rapid identification of *S. agalactiae* and the application of molecular typing methods can provide valuable epidemiological insights into mastitis caused by *S. agalactiae* in dairy farms. Our findings revealed a single clone with CPS type Ia and ST 103, indicating the transmission and circulation of this pathogen in herds with contagious behavior. Beta-lactam antimicrobials have proven to be effective and should be judiciously selected for a robust treatment protocol. Treating all quarters of infected cows is essential for the effective control and eradication of mastitis in the evaluated herds.

## Data availability statement

The original contributions presented in the study are included in the article/Supplementary material, further inquiries can be directed to the corresponding author.

## Ethics statement

All procedures have been reviewed and approved by the Institutional Biosafety Use protocol [Chulalongkorn University, Faculty of Veterinary Science] No. IBC 1931051.

## Author contributions

KA and SW designed the study, conducted statistical analyses, and drafted the manuscript. SW, TB, and SS performed laboratory analyses. SW assembled the data and interpreted the results. All authors contributed to the article and approved the submitted version.
