# Supplementary material for: Genotypic and antimicrobial susceptibility of Streptococcus agalactiae causing bovine mastitis in the central region of Thailand
Source: Front Vet Sci. 2023 Nov 9;10:1250436. doi: 10.3389/fvets.2023.1250436 (PMC10666187; doi:10.3389/fvets.2023.1250436)
Supplement: Supplementary file 1 [file Data_Sheet_1.pdf]

Supplementary Table 1. The 33 isolates selected for MLST typing.

ID	Herd	CowID	Quarter[1]	Types of mastitis[2]	Date of collection
SACU_13	A	6864	LF	1	12/18/2018
SACU_14	A	58338	RH	1	12/18/2018
SACU_17	A	58041	LF	1	12/18/2018
SACU_18	A	58041	LF	1	12/26/2018
SACU_20	A	58041	RF	2	12/26/2018
SACU_22	A	6864	LF	1	12/26/2018
SACU_23	A	58338	RH	1	12/26/2018
SACU_25	A	0102131	RF	2	7/19/2019
SACU_26	A	0102131	RH	1	7/19/2019
SACU_27	A	0102131	LF	2	7/19/2019
SACU_28	A	0102131	LH	1	7/19/2019
SACU_44	B	Makham	RH	2	16/8/2016
SACU_59	C	39	RF	2	5/7/2017
SACU_39	D	Yaya	RF	2	16/8/2016
SACU_40	D	Yaya	LH	2	8/3/2017
SACU_41	D	Yaya	RH	2	8/3/2017
SACU_93	E	Tia	LH	2	16/8/2016
SACU_67	F	Aumpun	RF	2	16/8/2016
SACU_68	F	Aumpun	LF	1	16/8/2016
SACU_69	F	Aumpun	RH	1	16/8/2016
SACU_70	F	Aumpun	LH	2	16/8/2016
SACU_37	G	Sangjang	RF	2	24/07/2017
SACU_49	h	Baitoey	LF	2	26/8/2017
SACU_87	H	Baitoey	LH	2	26/8/2017
SACU_31	I	Chamod	RF	2	26/08/2017
SACU_32	I	Chamod	LH	2	26/08/2017
SACU_66	J	Mudmee	RH	1	16/8/2016

SACU_86	J	Mudmee	LH	2	16/8/2016
SACU_47	K	Som	RH	2	26/8/2017
SACU_84	K	Som	RF	2	26/8/2017
SACU_96	L	Wan	LF	1	14/5/2019
SACU_97	L	Wan	LH	1	14/5/2019
SACU_73	M	Nanthana	RF	2	15/5/2019

[1] Quarter infected; LF, left front; LH, left-hind; RF, right front; RH, right hind

[2] Type of mastitis; 1, clinical mastitis; 2, subclinical mastitis

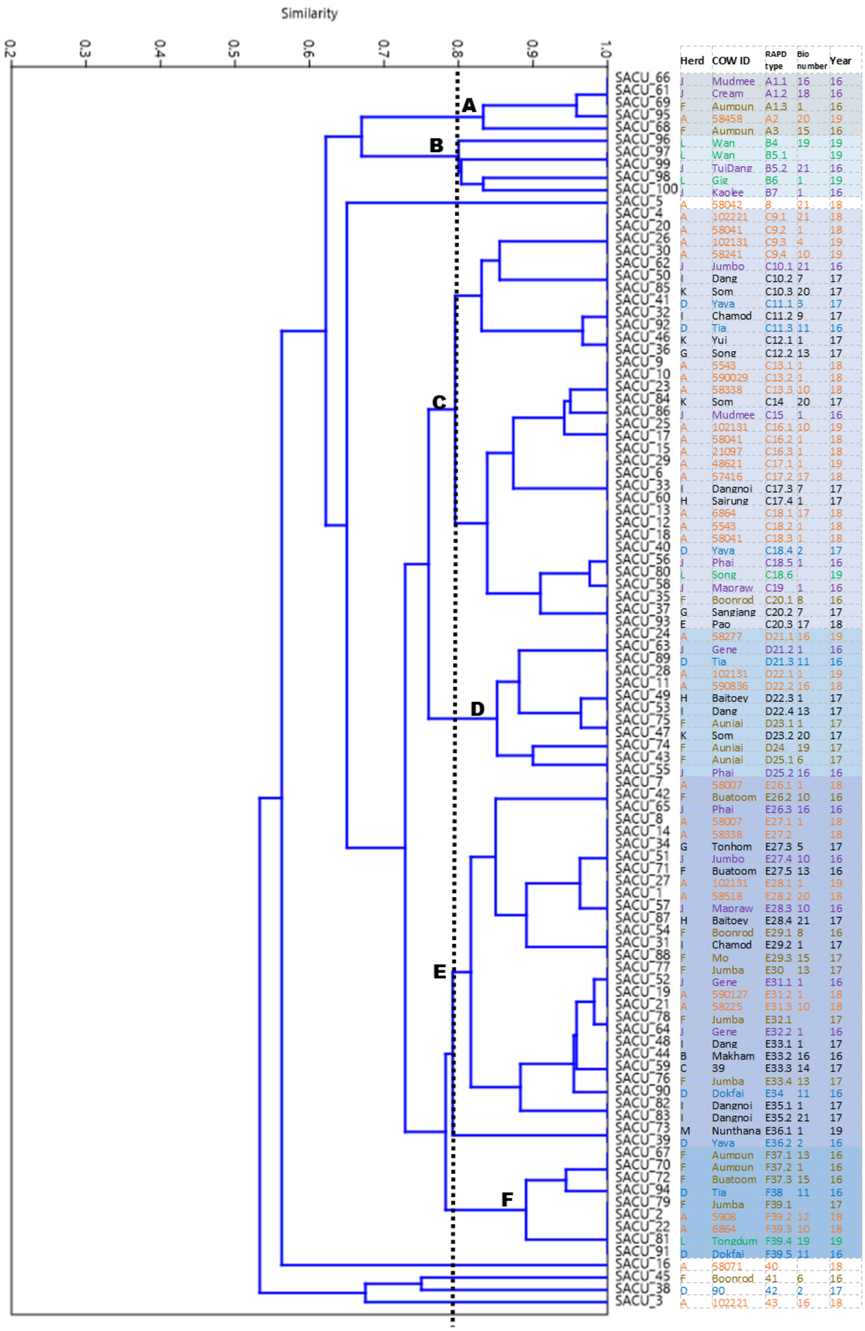

Herd	COW ID	RAPD type	Bio number	Year
J	Mudmee	A1.1	16	16
J	Cream	A1.2	18	16
F	Aumpun	A1.3	1	16
A	58458	A2	20	19
F	Aumpun	A3	15	16
L	Wan	B4	19	19
L	Wan	B5.1		19
J	TuiDang	B5.2	21	16
L	Gig	B6	1	19
J	Kaolee	B7	1	16
A	58042	8	21	18
A	102221	C9.1	21	18
A	58041	C9.2	1	18
A	102131	C9.3	4	19
A	58241	C9.4	10	19
J	Jumbo	C10.1	21	16
I	Dang	C10.2	7	17
K	Som	C10.3	20	17
D	Yaya	C11.1	3	17
I	Chamod	C11.2	9	17
D	Tia	C11.3	11	16
K	Yui	C12.1	1	17
G	Song	C12.2	13	17
A	5543	C13.1	1	18
A	590029	C13.2	1	18
A	58338	C13.3	10	18
K	Som	C14	20	17
J	Mudmee	C15	1	16
A	102131	C16.1	10	19
A	58041	C16.2	1	18
A	21097	C16.3	1	18
A	48621	C17.1	1	19
A	57416	C17.2	17	18
I	Dangnoi	C17.3	7	17
H	Sairung	C17.4	1	17
A	6864	C18.1	17	18
A	5543	C18.2	1	18
A	58041	C18.3	1	18
D	Yaya	C18.4	2	17
J	Phai	C18.5	1	16
L	Song	C18.6		19
J	Mapraw	C19	1	16
F	Boonrod	C20.1	8	16
G	Sangjang	C20.2	7	17
E	Pao	C20.3	17	18
A	58277	D21.1	16	19
J	Gene	D21.2	1	16
D	Tia	D21.3	11	16
A	102131	D22.1	1	19
A	590836	D22.2	16	18
H	Baitoey	D22.3	1	17
I	Dang	D22.4	13	17
F	Auniai	D23.1	1	17
K	Som	D23.2	20	17
F	Auniai	D24	19	17
F	Aunjai	D25.1	6	17
J	Phai	D25.2	16	16
A	58007	E26.1	1	18
F	Buatoom	E26.2	10	16
J	Phai	E26.3	16	16
A	58007	E27.1	1	18
A	58338	E27.2		18
G	Tonhom	E27.3	5	17
J	Jumbo	E27.4	10	16
F	Buatoom	E27.5	13	16
A	102131	E28.1	1	19
A	58518	E28.2	20	18
J	Mapraw	E28.3	10	16
H	Baitoey	E28.4	21	17
F	Boonrod	E29.1	8	16
I	Chamod	E29.2	1	17
F	Mo	E29.3	15	17
F	Jumba	E30	13	17
J	Gene	E31.1	1	16
A	590127	E31.2	1	18
A	58225	E31.3	10	18
F	Jumba	E32.1		17
J	Gene	E32.2	1	16
I	Dang	E33.1	1	17
B	Makham	E33.2	16	16
C	39	E33.3	14	17
F	Jumba	E33.4	13	17
D	Dokfai	E34	11	16
I	Dangnoi	E35.1	1	17
I	Dangnoi	E35.2	21	17
M	Nunthana	E36.1	1	19
D	Yaya	E36.2	2	16
F	Aumpun	F37.1	13	16
F	Aumpun	F37.2	1	16
F	Buatoom	F37.3	15	16
D	Tia	F38	11	16
F	Jumba	F39.1		17
A	5908	F39.2	12	18
A	6864	F39.3	10	18
L	Tongdum	F39.4	19	19
D	Dokfai	F39.5	11	16
A	58071	40		18
F	Boonrod	41	6	16
D	90	42	2	17
A	102221	43	16	18

Supplementary Figure 1. A dendrogram was constructed based on these RAPD profiles, and a similarity analysis revealed 43 distinct RAPD types among the 100 *S. agalactiae* isolates.
The blue shades (#D6DDE4, #DDEBF7, #D8DFEF, #BAD3E8, #B4C6E8, #9BC2E6 represent cluster A, B, C, D, E and F, respectively.

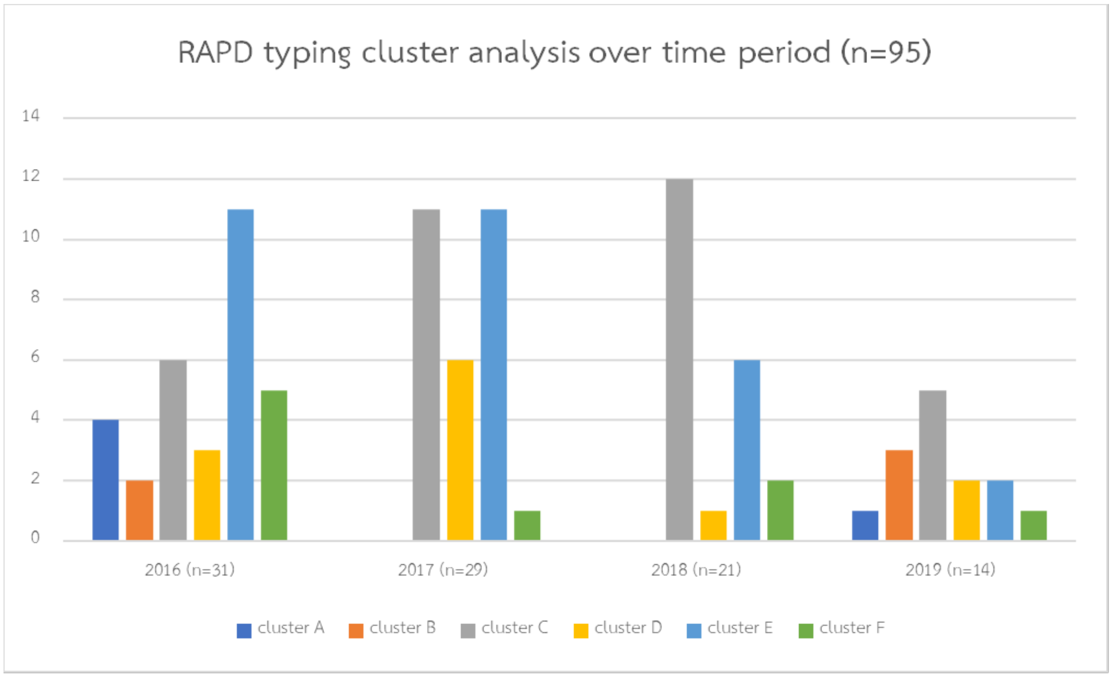

Supplementary Figure 2. The distribution of RAPD cluster types during the collection period (2016–2019). Within our collection of *S. agalactiae* isolates, clusters C (n = 34), D (n = 12), E (n = 30), and F (n = 9) were consistently identified throughout 2016–2019. However, clusters A and B (n = 5) were observed only in 2016 and 2019.

Supplementary Table 2. RAPD-PCR clustering of *Streptococcus agalactiae* isolates collected from 2016 to 2019 in 13 herds. Strains with similarity ≥80% were considered in the same cluster.

Cluster	Isolates	Herd	Cow ID	Date of collection	RAPD type
A	SACU_66	J	Mudmee	16/08/2016	A1.1
	SACU_61	J	Cream	16/08/2016	A1.2
	SACU_69	F	Aumpun	16/08/2016	A1.3
	SACU_95	A	58458	20/08/2019	A2
	SACU_68	F	Aumpun	16/08/2016	A3
B	SACU_96	L	Wan	14/05/2019	B4
	SACU_97	L	Wan	14/05/2019	B5.1
	SACU_99	J	TuiDang	16/08/2016	B5.2
	SACU_98	L	Gig	14/05/2019	B6
	SACU_100	J	Kaolee	16/08/2016	B7
	SACU_5	A	58042	31/10/2018	8
C	SACU_4	A	102221	31/10/2018	C9.1
	SACU_20	A	58041	26/12/2018	C9.2
	SACU_26	A	102131	19/07/2019	C9.3
	SACU_30	A	58241	19/07/2019	C9.4
	SACU_62	J	Jumbo	16/08/2016	C10.1
	SACU_50	I	Dang	26/08/2017	C10.2
	SACU_85	K	Som	26/08/2017	C10.3
	SACU_41	D	Yaya	3/8/2017	C11.1
	SACU_32	I	Chamod	26/08/2017	C11.2
	SACU_92	D	Tia	16/08/2016	C11.3
	SACU_46	K	Yui	26/08/2017	C12.1
	SACU_36	G	Song	24/07/2017	C12.2
	SACU_9	A	5543	31/10/2018	C13.1
	SACU_10	A	590029	31/10/2018	C13.2
	SACU_23	A	58338	26/12/2018	C13.3
	SACU_84	K	Som	26/08/2017	C14
	SACU_86	J	Mudmee	16/08/2016	C15
	SACU_25	A	102131	19/07/2019	C16.1
	SACU_17	A	58041	18/12/2018	C16.2
	SACU_15	A	21097	18/12/2018	C16.3
	SACU_29	A	48621	19/07/2019	C17.1
	SACU_6	A	57416	31/10/2018	C17.2
	SACU_33	I	Dangnoi	26/08/2017	C17.3
	SACU_60	H	Sairung	26/08/2017	C17.4
	SACU_13	A	6864	18/12/2018	C18.1
	SACU_12	A	5543	31/10/2018	C18.2
	SACU_18	A	58041	26/12/2018	C18.3
	SACU_40	D	Yaya	3/8/2017	C18.4

	SACU_56	J	Phai	16/08/2016	C18.5
	SACU_80	L	Song	14/05/2019	C18.6
	SACU_58	J	Mapraw	16/08/2016	C19
	SACU_35	F	Boonrod	16/08/2016	C20.1
	SACU_37	G	Sangjang	24/07/2017	C20.2
	SACU_93	E	Pao	15/08/2018	C20.3
D	SACU_24	A	58277	19/07/2019	D21.1
	SACU_63	J	Gene	16/08/2016	D21.2
	SACU_89	D	Tia	16/08/2016	D21.3
	SACU_28	A	102131	19/07/2019	D22.1
	SACU_11	A	590836	31/10/2018	D22.2
	SACU_49	H	Baitoey	26/08/2017	D22.3
	SACU_53	I	Dang	26/08/2017	D22.4
	SACU_75	F	Aunjai	26/01/2017	D23.1
	SACU_47	K	Som	26/08/2017	D23.2
	SACU_74	F	Aunjai	26/01/2017	D24
	SACU_43	F	Aunjai	26/01/2017	D25.1
	SACU_55	J	Phai	16/08/2016	D25.2
E	SACU_7	A	58007	31/10/2018	E26.1
	SACU_42	F	Buatoom	16/08/2016	E26.2
	SACU_65	J	Phai	16/08/2016	E26.3
	SACU_8	A	58007	31/10/2018	E27.1
	SACU_14	A	58338	18/12/2018	E27.2
	SACU_34	G	Tonhom	13/08/2017	E27.3
	SACU_51	J	Jumbo	16/08/2016	E27.4
	SACU_71	F	Buatoom	16/08/2016	E27.5
	SACU_27	A	102131	19/07/2019	E28.1
	SACU_1	A	58518	26/09/2018	E28.2
	SACU_57	J	Mapraw	16/08/2016	E28.3
	SACU_87	H	Baitoey	26/08/2017	E28.4
	SACU_54	F	Boonrod	16/08/2016	E29.1
	SACU_31	I	Chamod	26/08/2017	E29.2
	SACU_88	F	Mo	15/02/2017	E29.3
	SACU_77	F	Jumba	26/01/2017	E30
	SACU_52	J	Gene	16/08/2016	E31.1
	SACU_19	A	590127	26/12/2018	E31.2
	SACU_21	A	58225	26/12/2018	E31.3
	SACU_78	F	Jumba	15/02/2017	E32.1
	SACU_64	J	Gene	16/08/2016	E32.2
	SACU_48	I	Dang	26/08/2017	E33.1
	SACU_44	B	Makham	16/08/2016	E33.2
	SACU_59	C	39	7/5/2017	E33.3
	SACU_76	F	Jumba	26/01/2017	E33.4
	SACU_90	D	Dokfai	16/08/2016	E34

	SACU_82	I	Dangnoi	26/08/2017	E35.1
	SACU_83	I	Dangnoi	26/08/2017	E35.2
	SACU_73	M	Nunthana	15/05/2019	E36.1
	SACU_39	D	Yaya	16/08/2016	E36.2
F	SACU_67	F	Aumpun	16/08/2016	F37.1
	SACU_70	F	Aumpun	16/08/2016	F37.2
	SACU_72	F	Buatoom	16/08/2016	F37.3
	SACU_94	D	Tia	16/08/2016	F38
	SACU_79	F	Jumba	15/02/2017	F39.1
	SACU_2	A	5908	26/09/2018	F39.2
	SACU_22	A	6864	26/12/2018	F39.3
	SACU_81	L	Tongdum	14/05/2019	F39.4
	SACU_91	D	Dokfai	16/08/2016	F39.5
	SACU_16	A	58071	18/12/2018	40
	SACU_45	F	Boonrod	16/08/2016	41
	SACU_38	D	90	03/08/2017	42
	SACU_3	A	102221	31/10/2018	43
